# Chiari Malformation Type 1 in *EPAS1*-Associated Syndrome

**DOI:** 10.3390/ijms20112819

**Published:** 2019-06-10

**Authors:** Jared S. Rosenblum, Dominic Maggio, Ying Pang, Matthew A. Nazari, Melissa K. Gonzales, Ronald M. Lechan, James G. Smirniotopoulos, Zhengping Zhuang, Karel Pacak, John D. Heiss

**Affiliations:** 1 Neuro-Oncology Branch, National Cancer Institute, National Institutes of Health, Bethesda, MD 20892, USA; jared.rosenblum@nih.gov; 2 Surgical Neurology Branch, National Institute of Neurological Disorders and Stroke, National Institutes of Health, Bethesda, MD 20892, USA; dominic.maggio@nih.gov; 3 Section on Medical Neuroendocrinology, Eunice Kennedy Shriver National Institute of Child Health and Human Development, National Institutes of Health, Bethesda, MD 20892, USA; Ying.pang@nih.gov (Y.P.); melissa.gonzales@nih.gov (M.K.G.); 4 Department of Internal Medicine and Pediatrics, Georgetown University Medical Center, Washington, DC 20007, USA; matt.a.nazari@gmail.com; 5 Division of Endocrinology, Diabetes & Metabolism, Tufts Medical Center, Boston, MA 02111, USA; rlechan@tuftsmedicalcenter.org; 6 Radiology, George Washington University, Washington, DC 20052, USA; medpixman@gmail.com; 7 MedPix^®^ National Library of Medicine, 8600 Rockville Pike Bethesda, MD 20894, USA

**Keywords:** Chiari malformation type I, Pacak-Zhuang syndrome, EPAS1, HIF-2α

## Abstract

A syndrome of multiple paragangliomas/pheochromocytomas, somatostatinoma, and polycythemia due to somatic mosaic gain-of-function mutation of *EPAS1*, encoding HIF-2α, was previously described. HIF-2α has been implicated in endochondral and intramembranous ossification. Abnormal bone growth of the skull base may lead to Chiari malformation type I. We report two cases of *EPAS1* gain-of-function mutation syndrome with Chiari malformation and developmental skull base anomalies. Patients were referred to the Section on Medical Endocrinology, *Eunice Kennedy Shriver* NICHD, NIH for evaluation of recurrent and metastatic paragangliomas or pheochromocytoma. The syndrome was confirmed genetically by identification of the functional *EPAS1* gain-of-function mutation in the resected tumors and circulating leukocytes. Both patients were confirmed for characteristics of *EPAS1* gain-of-function mutation syndrome by complete blood count (CBC), plasma biochemistry, and computed tomography (CT) of the abdomen and pelvis. Chiari malformation type I and abnormal bony development of the posterior fossa was found on MRI and CT of the head. The present study implicates *EPAS1* mutations in abnormal posterior fossa development resulting in Chiari malformation type I.

## 1. Introduction

Chiari malformation type I is characterized by caudal displacement of the cerebellar tonsils through the foramen magnum of at least 5 mm in adults [1]. Several studies have implicated shortened posterior fossa bones and reduced posterior fossa volume in the development of Chiari malformation type I [2]. The pathways that lead to Chiari I malformation development are multifactorial, complex, and not fully elucidated [3]. While Chiari malformation type I has been found to co-occur with genetic syndromes such as achondroplasia, to date, no causative gene mutation has been identified for most patients diagnosed with Chiari I malformation [4,5]. Further, Chiari malformation type I has not been reported to be associated with systemic cancer, although posterior fossa tumors may produce cerebellar tonsillar herniation similar to seen in Chiari I malformation.

*EPAS1*, encoding hypoxia-inducible factor-2α (HIF-2α), is active in regions of hypoxia in development, including endochondral and intramembranous ossification [6,7]. Posterior fossa bones are formed by endochondral ossification [8]. Mosaic gain-of-function *EPAS1* mutations have been found to cause a syndrome encompassing multiple paragangliomas/pheochromocytomas, somatostatinoma, and polycythemia, also called Pacak-Zhuang syndrome [9]. Herein, we report two patients with *EPAS1* gain-of-function mutation syndrome, Chiari malformation type I and other developmental anomalies of the posterior fossa and spine. 

## 2. Results

Both patients were found to be negative for mutations in erythropoietin, erythropoietin receptor, HIF-1α, janus kinase 2, prolyl hydroxylase, succinate dehydrogenase B/C/D, and von Hippel-Lindau genes in circulating leukocytes and resected tumor tissues (Table 1). This served as genetic confirmation of isolated *EPAS1*-gain-of-function syndrome and allowed the evaluation of HIF-2α in the absence of common somatic mutations causing alterations in the hypoxia pathway resulting in paraganglioma/pheochromocytoma.

### 2.1. Summary of Cases

#### 2.1.1. Case 1

##### History of Present Illness

A 50-year-old woman presented to our institution 14 years ago with a past medical history significant for polycythemia diagnosed at age 7. An obstructive mass in the head of the pancreas was found and resected at age 36, using the Whipple procedure. Blood pressure was labile during the procedure. Histology confirmed duodenal ampullary somatostatinoma and additional multiple abdominal paragangliomas. She was at that time referred to our institution for investigation of suspected metastatic paraganglioma. Blood chemistries (Table 1) and ^18^F-fluorodeoxyglucose positron emission tomography/computed tomography (^18^FDG PET/CT) confirmed metastatic and/or additional paragangliomas with foci present in the left para-aortic region at the level of the kidney and throughout the abdomen, corresponding to ^123^I-metaiodobenzylguanidine (^123^I-MIBG) activity on subsequent scan. She underwent ^131^I-MIBG-directed radiotherapy and resection of these tumors at an outside institution. Identification of the *EPAS1* gain-of-function mutation in the resected tumor tissues and circulating leukocytes confirmed the underlying syndrome. At present, tumor burden is stable, polycythemia has resolved although erythropoietin levels are mildly elevated, and the patient is asymptomatic. 

##### Imaging Findings

**Figure 1:**
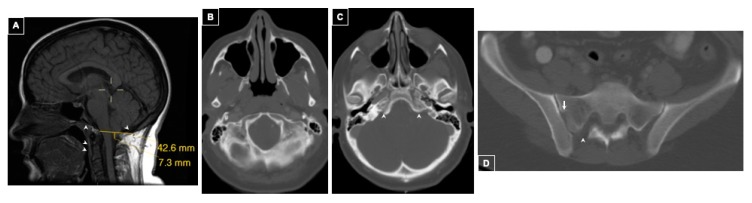
Patient 1 Intracranial and Spinal Imaging. Panel (**A**): Mid-sagittal T1-weighted sequence shows lowest tonsillar position of 7 mm through the foramen magnum. Narrow underdeveloped clivus and occipital bone as well as uncalcified synchondroses of the odontoid are appreciated (arrowheads). Panel (**B**): Axial CT of the head revealed abnormal ossification of the occipital bone. Panel (**C**): Axial CT of the head showing uncalcified petroclival synchondrosis (arrowheads). Panel (**D**): Axial CT of lumbo-sacral spine shows spina bifida occulta of S1 (arrowhead) and segmentation anomaly of the sacral ala (arrow).

Imaging of the chest, abdomen, and pelvis showed stable tumor size and occult sacral dysraphism and segmentation defect. l-3,4-dihdroxy-6-[^18^F]fluorophenylalanine (^18^F-FDOPA)-PET/CT scan revealed focal tracer uptake at the skull base consistent with a glomus jugulare tumor (probably paraganglioma) and tracer uptake within several lumbar vertebral bodies. CT and MRI of the head were diagnostic of Chiari malformation type I with tonsillar displacement 7 mm below the foramen magnum (Figure 1A). Reduced ossification of the occipital bone and uncalcified petroclival synchondrosis were evident on CT (Figure 1B–D). 

##### Family History

No family members have history of pheochromocytoma/paraganglioma, polycythemia, thyroid disease, parathyroid disease, adrenal disease, or history of Chiari malformation. Her mother died from lymphoma at age 62. Her father died of bladder cancer at age 75. 

#### 2.1.2. Case 2

##### History of Present Illness

A 29-year-old male presented to our institution with a past medical history significant for polycythemia diagnosed at 28 months by routine phlebotomy. At age 14, a left chest wall tumor was found at level of the 4th rib on CT scan performed for evaluation of hypertension and syncopal episodes. Blood chemistries (Table 1) and histopathology of three resected chest wall masses at age 17 confirmed paragangliomas. He was referred to our institution for genetic evaluation after resection of multiple paraortic and lumbar paragangliomas. Somatic mosaicism of *EPAS1* gain-of-function was confirmed in the resected tumors and circulating leukocytes. His follow-up included routine phlebotomy for polycythemia and measurement of plasma and urinary metanephrines and imaging for tumor surveillance and detection. 

##### Imaging Findings 

**Figure 2:**
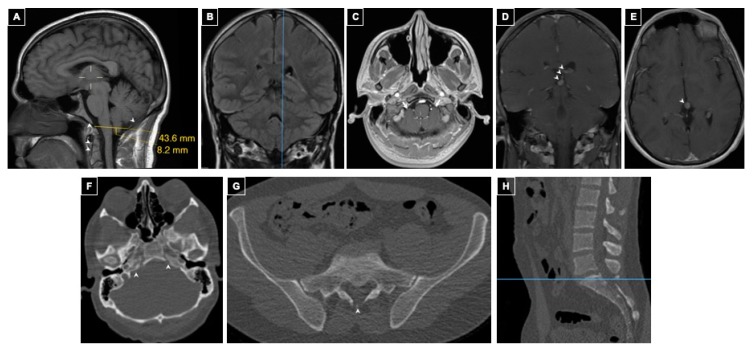
Patient 2 Intracranial and Spinal Imaging. Panels (**A**–**E**)*:* MRI of the brain. Panel (**A**): Sagittal post-contrast T1-weighted sequence shows the left cerebellar tonsil at the lowest tonsillar position, 8 mm through the foramen magnum. Narrow, underdeveloped clivus and occipital bone as well as uncalcified synchondroses of the odontoid are also seen (arrowheads). Panel (**B**): Coronal T1-weighted sequence demonstrating the location of the measurement in A. Panel (**C**): Axial post-contrast T1-weighted sequence showing crowding of the brainstem by the cerebellar tonsils in the foramen magnum (arrows). Panel (**D**): Coronal T1-weighted venous phase post-contrast sequence shows a developmental venous anomaly of the Vein of Galen confluens draining the choroid plexus through the velum interpositum (arrowheads). Panel (**E**): Axial T1-weighted venous phase post-contrast sequence showing the anomalous Vein of Galen confluens. Panel (**F**): Axial CT of the head showing petroclival dysraphism or uncalcified petroclival synchondrosis (arrowheads). Panel (**G**): Axial CT of lumbo-sacral spine shows occult dysraphism of S1 (arrowhead) and sacral segmentation anomalies (arrows). Panel (**H**): Sagittal lumbar CT demonstrating the level of the image shown in G.

^18^F-FDOPA scan revealed a focus of tracer uptake in the right lower lobe and six retroperitoneal foci in the aortocaval and right paracaval regions; the para-aortic lesions were also positive on ^68^Ga- DOTATATE PET/CT. MRI of the brain with contrast performed for evaluation of metastatic disease detected Chiari type I malformation with tonsillar displacement 8 mm below the foramen magnum (Figure 2A–C). A developmental venous anomaly of the Vein of Galen confluens and choroid plexus involving the velum interpositum was also found (Figure 2D–E). Sacral dysraphism was also present (Figure 2F–H). Imaging follow up over several years in patient 2 demonstrated that the Chiari I malformation was stable.

## 3. Discussion

The cases of Chiari I malformation associated with *EPAS1* syndrome provide possible unique insight into the development of Chiari malformation. We are currently systematically characterizing *EPAS1* syndrome, which was originally described as a constellation of paraganglioma/pheochromocytoma, polycythemia, and somatostatinoma [6]. Eight of our 11 patients with this syndrome have been evaluated by CT and/or MRI of the head and CT of the spine. Of the 8 studied patients, 7 have cerebellar tonsillar ectopia (1–8 mm) and occult sacral dysraphism. We chose to report the two patients presented herein separately because their extent of tonsillar ectopia fulfilled the imaging diagnostic criteria for Chiari I malformation. The patients have not developed symptoms of Chiari I malformation, however. 

Chiari I malformation is commonly associated with a small posterior fossa and low insertion of the tentorium [1,2]. Brain anomalies often associated with Chiari II malformation, including enlarged fused massa intermedia and beaked tectum, were not present in these two cases. Although dysraphism and segmentation anomalies of the sacrum were present in both cases, there was no primary neural tube defect of the spinal cord, which is usually associated with Chiari II malformation [2]. While incidental spina bifida occulta has a frequency of about 3 per 10,000 births according to the CDC [11], the finding of dysraphic processes and Chiari malformation type I in these patients suggests a common mechanism. 

Regulation of HIF-2α is critical to proper ossification of cartilage [6]. The bony spina bifida occulta without involvement of the spinal cord that is present in these patients suggests inadequate closure of the dorsal mesoderm following closure of the neural tube at the posterior neuropore. Thus, this suggests that the gain-of-function mutation in HIF-2α leads to the persistence of cartilaginous structures due to delayed closure of the dorsal mesoderm or lack of endochondral ossification in bony growth centers of the sacral spinous processes and lamina. The same mechanism may be true of the clivus and occipital bone, which also undergo endochondral ossification [8]. This is supported by the thin appearance of the clivus and occipital bone in both patients.

Chiari I malformation has been attributed to a small posterior fossa that is unable to contain the neural elements, leading to downward tonsillar herniation into the spinal canal [12,13,14]. Patient 1 in our study had a crowded posterior fossa. However, there is a spacious subtype of Chiari malformation type I in which tonsillar displacement occurs even though the posterior fossa size is normal [15]. Patient 2 had the spacious subtype of Chiari malformation type I. Neither patient had abnormally short clivus or supraocciput, nor platybasia or basilar invagination (Table 1). However, both patients had uncalcified synchondroses of the clivus, supraocciput, and odontoid as well as spina bifida occulta, supporting impaired developmental ossification of these structures. Despite uncalcified synchondroses of the clivus and supraocciput, the skull base was structurally sound as shown by the absence of basilar impression (Table 1). An alternate explanation for the disproportion of the cerebellum and posterior fossa volume was that the mutation resulted in enlargement of the cerebellum.

The patients reported have incidental Chiari I malformation, a term used to describe patients with MRI findings diagnostic for Chiari I malformation but no associated symptoms of the condition. Some patients with incidental Chiari I malformation later develop symptoms of the condition either spontaneously or after physical trauma. The appearance of incidental Chiari malformation and reduced bony development of the spine and skull base in our patients with gain-of-function *EPAS1* mutation suggests that persistent hypoxic signaling resulted in incomplete mesenchymal development that manifested as 1) reduced ossification of posterior fossa bones and, secondarily, Chiari I malformation, 2) impaired development of the sacrum leading to spina bifida occulta of S1 and segmentation anomalies of the sacral ala, and 3) impaired development of other brain structures resulting in asymptomatic brain vascular anomalies [16]. 

## 4. Materials and Methods 

### 4.1. Study Oversight

This study (Protocol, 00-CH-0093: Diagnosis, Pathophysiology, and Molecular Biology of Pheochromocytoma and Paraganglioma) was approved by the institutional review board of the *Eunice Kennedy Shriver* National Institute of Child Health and Human Development, NIH. The study was approved in the continuing review on was approved in the continuing review on 12 December 2018. Both patients provided written informed consent.

### 4.2. Patient Selection and Evaluation

Typical features of the syndrome, including polycythemia, paraganglioma/pheochromocytoma, and duodenal somatostatinoma, were confirmed in the selected patients by complete blood count (CBC), erythropoietin (EPO), plasma and/or urine catecholamines/metanephrines, somatostatin, computed tomography (CT), positron emission tomography (PET)/CT, and/or MRI of the neck, chest, abdomen, and pelvis (Table 1) and pathology. Patients underwent MRI of the brain. Bony anatomy of the calvarium, skull base, and spine was evaluated on CT reconstructed images. 

### 4.3. Patient EPAS1 Mutation Analysis

Genomic DNA was extracted from patient leukocytes and resected tumor tissues via NucleoSpin Tissue Kit (Macherey-Nagel, Bethlehem, PA, USA). *EPAS1* exons were amplified by means of polymerase-chain-reaction (PCR) assay. Primer sets for exon amplification have been previously described [9]. The DNA sequence of each exon was determined by forward and reverse sequencing [9].

### 4.4. Measurements

Tonsillar displacement was measured according to the National Institute of Neurological Disorders and Stroke Common Data Elements for Chiari I Malformation (http://www.commondataelements.ninds.nih.gov, accessed: 2 February 2019). Tentorial Angle was measured as described by Kao et al. [17]. Measurements of tonsillar displacement, posterior fossa height, cervicomedullary kinking (Klaus index height), pontomedullary height, and platybasia were performed as previously described [10]. Basilar invagination was defined as the odontoid tip lying at least 5 mm above the McGregor line [18]. 

## 5. Conclusions

Herein, we describe two patients with *EPAS1-*gain-of-function (Pacak-Zhuang) syndrome and features of incidental Chiari I malformation, i.e., greater than 5 mm tonsillar herniation below the foramen magnum. While the posterior fossa in these patients is not small, the boney development appears abnormal. The reduced boney development of the spine and skull base in these patients with gain-of-function mutation in *EPAS1* suggests that persistent hypoxic signaling results in incomplete mesenchymal development. This report implicates mutations in *EPAS1*, encoding HIF-2α, in the development of Chiari malformation type I.

## Figures and Tables

**Table 1:** *EPAS1 G*ain-of-Function Syndrome Patient Characteristics.

	Patient	
Age at Onset of Diagnosed Condition (years)		
Polycythemia (HCT >51.0%)	7	2.3
EPO (mIU/mL)	165.0 (3.7–31.5)	101 (2.6–18.5)
PGL/PHEO	36	14
Ampullary Somatostatinoma	36	−
Mutation Analysis—Tumors and Circulating Leukocytes		
*EPAS1* Gain-of-Function Mutation	Y532C	D539N
*EPOR, HIF-1*α,* JAK2, PHD1/2, SDHB/C/D, VHL*	Negative	Negative
Clinical Characteristics		
Blood Pressure (mm Hg)		
At Presentation to NIH	111/69	141/85
Heart Rate (bpm)	71	75
Chiari Malformation Features		
Lowest Cerebellar Tonsillar Position (mm)	7	8
Position of Right Cerebellar Tonsil (mm)	4	6
Position of Left Cerebellar Tonsil (mm)	7	8
Shape of Cerebellar Tonsils	Pegged	Round
Ventral CSF Space (12 ± 2.3 mm) [8]	4	9
Dorsal CSF Space (19 ± 2.3 mm) [8]	0	2
Pontomedullary Junction to Foramen Magnum (19 ± 3 mm) [10]	14	15
Tectal Beaking	No	No
Supraoccipital Bone Length (41 ± 5 mm) [10]	35	46
Clival Length (43.4 ± 4.4 mm) [10]	41	44
Tentorial Angle (27–52°) [10]	50.40	63.07
Klaus Index Height (38.0 ± 5 mm) [10]	49	51
Boogaard Angle (133.8 ± 6.5°) [10]	130	114
McGregor Line (<4.5 mm)	<0	<0
Posterior Fossa Height (32 ± 3 mm) [10]	32	39
Uncalcified Petroclival Synchondrosis	Yes	Yes
Sacral spina bifida occulta	Yes	Yes

Patient Characteristics. EPO, erythropoietin; EPOR, erythropoietin receptor; HIF-1α, hypoxia-inducible factor 1α. JAK2, janus kinase 2; PHD prolyl hydroxylase; PGL/PHEO, paraganglioma/pheochromocytoma; SDHB/C/D, succinate dehydrogenase B/C/D; VHL, von Hippel-Lindau.

## Abbreviations

HIF-2α	Hypoxia-inducible factor 2α
^18^FDG PET/CT	^18^F-fluorodeoxyglucose positron emission tomography/computed tomography
^123^I-MIBG	^123^I-metaiodobenzylguanidine
EPO	Erythropoietin
EPOR	Erythropoietin receptor
HIF-1α	Hypoxia-inducible factor 1α
JAK2	Janus kinase 2
PHD	Prolyl hydroxylase
PGL/PHEO	Paraganglioma/pheochromocytoma
SDHB/C/D	Succinate dehydrogenase B/C/D
VHL	von Hippel-Lindau
^18^F-FDOPA)-PET/CT	l-3,4-dihdroxy-6-[^18^F]fluorophenylalanine PET/CT scan

## References

[1] Bano S., Chaudhary S., Yadav S., Bright P. (2012). Congenital Malformation of the Brain, Neuroimaging—Clinical Applications.

[2] McLone D.G., Knepper P.A. (1989). The cause of Chiari II malformation: A unified theory. Pediatr. Neurosci..

[3] Shoja M.M., Johal J., Oakes W.J., Tubbs R.S. (2018). Embryology and pathophysiology of the Chiari I and II malformations: A comprehensive review. Clin. Anat..

[4] Kniffin C.L., McKusick V.A. OMIM-Online Mendelian Inheritance in Man: Chiari Malformation Type, I. Published 5/12/1992. Updated 10/07/2016. https://www.omim.org/entry/118420#title.

[5] Urbizu A., Khan T.N., Ashley-Koch A.E. (2016). Genetic dissection of Chiari malformation type I using endophenotypes and stratification. J. Rare Dis. Res. Treat..

[6] Dunwoodie S.L. (2009). The Role of Hypoxia in Development of the Mammalian Embryo. Dev. Cell.

[7] Lee S.Y., Park K.H., Yu H.G., Kook E., Song W.H., Gyuseok L., Koh J.T., Shin H.I., Choi J.Y., Huh Y.H. (2019). Controlling hypoxia-inducible factor-2α is critical for maintaining bone homeostasis in mice. Bone Res..

[8] Bess S., Varma V., Akbarnia B.A., Yazici M., Thompson G.H. (2010). Embryology and Anatomy: Spine/Spinal Cord. The Growing Spine: Management of Spinal Disorders in Young Children.

[9] Zhuang Z., Yang C., Lorenzo F., Merino M., Fojo T., Kebebew E., Popovic V., Stratakis C.A., Prchal J.T., Pacak K. (2012). Somatic HIF2A Gain-of-Function Mutations in Paraganglioma with Polycythemia. N. Engl. J. Med..

[10] Bogdanov E.I., Heiss J.D., Mendelevich E.G., Mikhaylov I.M., Haass A. (2004). Clinical and neuroimaging features of “idiopathic” syringomyelia. Neurology.

[11] Centers for Disease Control and Prevention Data and Statistics on Spina Bifida|CDC. Washington, DC, USA: Center for Disease Control, 13 September 2018. https://www.cdc.gov/ncbddd/spinabifida/data.html.

[12] Marin-Padilla M. (1979). Notochordal-basichondrocranium relationships: Abnormalities in experimental axial skeletal (dysraphic) disorders. J. Embryol. Exp. Morphol..

[13] Marina-Padilla M., Marina-Padilla T.M. (1981). Morphogenesis of experimentally induced Arnold-Chiari malformation. J. Neurol Sci..

[14] Nishikawa M., Sakamoto H., Hakuba A., Nakanishi N., Inoue Y. (1997). Pathogenesis of Chiari malformation: A morphometric study of the posterior cranial fossa. J. Neurosurg..

[15] Taylor D.G., Mastorakos P., Jane J.A., Oldfield E. (2017). Two distinct populations of Chiari I malformation based on presence or absence of posterior fossa crowdedness on magnetic resonance imaging. J. Neurosurg..

[16] Ogul H., Kantarci M. (2017). Unusual Association: Cerebral Arteriovenous Malformation and Chiari Type I Malformation. J. Craniofac. Surg..

[17] Kao S.C., Waziri M.H., Smith W.L., Sato Y., Yuh W.T., Franken E.A. (1989). MR imaging of the craniovertebral junction, cranium, and brain in children with achondroplasia. Am. J. Roentgenol..

[18] Pinter N.K., McVige J., Mechtler L. (2016). Basilar invagination, basilar impression, and platybasia: Clinical and imaging aspects. Curr. Pain Headache Rep..

